# The Expression of *TaRca2-α* Gene Associated with Net Photosynthesis Rate, Biomass and Grain Yield in Bread Wheat (*Triticum aestivum* L.) under Field Conditions

**DOI:** 10.1371/journal.pone.0161308

**Published:** 2016-08-22

**Authors:** Iqbal Saeed, Daoura Goudia Bachir, Liang Chen, Yin-Gang Hu

**Affiliations:** 1 State Key Laboratory of Crop Stress Biology for Arid Areas, College of Agronomy, Northwest A&F University, Yangling, Shaanxi, P.R. China; 2 NIFA, PO Box 446, Tarnab, Peshawar, KP, Pakistan; 3 Institute of Water Saving Agriculture in Arid Regions of China, Yangling, Shaanxi, 712100, China; Saint Mary's University, CANADA

## Abstract

Improvement in activation of Rubisco by Rubisco activase can potentially enhance CO_2_ assimilation and photosynthetic efficiency in plants. The three homoeologous copies of *TaRca2-α* were identified on chromosomes *4AL*, *4BS* and *4DS* (*TaRca2-α-4AL*, *TaRca2-α-4BS*, and *TaRca2-α-4DS*) in bread wheat. Expression patterns of the three copies at heading (Z55), anthesis (Z67) and grain-filling (Z73) stages were investigated through qRT-PCR analyses in a panel of 59 bread wheat genotypes and their effects on net photosynthesis rate (Pn), biomass plant^-1^ (BMPP) and grain yield plant^-1^ (GYPP) were further explored. Different but similar expression patterns were observed for the three copies of *TaRca2-α* at the three growth stages with highest expression at grain-filling stage. *TaRca2-α-4BS* expressed higher at the three stages than *TaRca2-α-4AL* and *TaRca2-α-4DS*. The 59 genotypes could be clustered into three groups as high (7 genotypes), intermediate (41 genotypes) and low (11 genotypes) expression based on the expression of the three copies of *TaRca2-α* at three growth stages. Significant variations (*P*<0.01) were observed among the three groups of bread wheat genotypes for Pn, BMPP and GYPP. Generally, the genotypes with higher *TaRca2-α* expression also showed higher values for Pn, BMPP and GYPP. The expressions of the three copies of *TaRca2-α* at heading, anthesis and grain-filling stages were positively correlated with Pn, BMPP and GYPP (*P*<0.01) with stronger association for *TaRca2-α-4BS* at grain-filling stage. These results revealed that the expression of *TaRca2-α* contribute substantially to Pn, BMPP and GYPP, and suggested that manipulating *TaRca-α* expression may efficiently improve Pn, BMPP and GYPP in bread wheat and detecting *TaRca-α* expression levels with emphasis on *TaRca2-α-4BS* may be a positive strategy for selection in improving photosynthetic efficiency and grain yield of bread wheat.

## Introduction

Wheat is the cereal of choice globally and is a source of about one-fifth of the total calories consumed by the world’s population [1], and is planted over 220 Mha of land throughout the world [2]. Although, Green revolution technologies have helped to a reasonable extent to enhance overall wheat productivity [3], meeting the demand of the fast-growing global population is a challenging task [4]. In order to feed the future population, emphasis needs to be concentrated on key traits related to plant productivity in the context of prevailing environmental conditions instead of solely relying on conventional practices. Photosynthesis being the basic constituent part of plant productivity can be efficiently manipulated to improve the overall productivity of wheat crop. Furthermore, positive relationship of photosynthesis with yield [5] makes it a desirable trait to be selected and manipulated for the enhancement of wheat yield potential.

For efficient photosynthesis to occur, the central role is played by the enzyme Rubisco (Ribulose-1,5-bisphosphate carboxylase/oxygenase), which is capable of catalyzing net fixation of inorganic carbon into organic molecules [6]. In spite of Rubisco’s enormous presence on earth, it has a lower efficiency due to slow catalytic turn-over rate [7]; consequently larger quantities of the enzyme are needed to ensure optimal photosynthesis. Furthermore, some natural inhibitory sugar phosphates attach to the active sites of Rubisco, rendering it inactive and hence incapable of fixing CO_2_ [8]. This also results in extra investments in nitrogen with environmental implications.

Rubisco needs to be converted from an inactive to catalytically active state for the efficient catalysis of photosynthetic assimilation of inorganic CO_2_ into organic compounds, and Rubsico activase (Rca) is the enzyme facilitating this conversion [9, 10]. Rca belongs to an extended AAA+ superfamily of ATPases, which is involved in various cellular activities [11] and regulates Rubisco’s activity by removing inhibitory sugar phosphates from Rubisco active sites using energy from ATP hydrolysis [12, 13]. Resultantly, the active sites of Rubisco are spontaneously carbamylated by CO_2_ and normal photosynthesis is supported. In most of plants, there are two isoforms of Rca, i.e. a large *α* isoform and a small *β* isoform with differences at the carboxy terminus [12] and also differing in maximal activity [14, 15] with *Rca-α* showing higher expression under most of the growth conditions [16]. The importance of *Rca-α* is also evident from the results of previous studies in different crops due its positive effect on plant productivity traits [16, 17] and therefore can also identify genotypes with improved phenotype under prevailing crop growth conditions. The success of crop plants depends on their final performance in the field where plants experience unpredictable changes in environmental conditions e.g. fluctuating irradiance, water deficit etc. Most studies concerning the effect of *Rca* on plant phenotype in wheat are conducted under preset conditions. Therefore, understanding the effect of *Rca-α* on wheat photosynthesis, biomass and grain yield under natural field conditions may provide a better strategy for improving overall productivity. Furthermore, it may also facilitate to detect the existing genetic variability among different wheat genotypes for photosynthesis, biomass and yield rated traits.

Being hexaploid, wheat has a very complex genome, each individual gene is potentially present in triplicate (A, B and D), and each homoeologue may express differentially and affect the phenotype in different manner. Investigation on the difference among the three copies not only helps in understanding the effect of a specific copy of a gene, but also reveals sequence diversity and facilitates to develop gene-based functional markers for marker-assisted breeding [18].

Based on the potential role of *TaRca2-*α on wheat photosynthesis and therefore grain yield especially under fluctuating environmental conditions, the present study was designed to investigate the expression patterns of *TaRca2-α* in flag leaves at three main growth stages (heading, anthesis and mid grain-filling) of bread wheat in a panel of 59 bread wheat genotypes grown under natural field conditions, and to test whether the expression levels of *TaRca2-α* in flag leaves associated with Pn, and BMPP and GYPP, and the contributions of the three individual copies of *TaRca2-α*.

## Materials and Methods

### Plant material and sowing

A panel of 59 winter wheat genotypes from two major wheat growing regions of China was used in the present study (Table 1), among those, 29 genotypes each were from the Northern and Huang-Huai Winter Wheat Regions, respectively and one genotype from Southwestern Winter Wheat Region. The genotypes were sown during 2013–14 and 2014–15 crop seasons on the experimental field at the Northwest A&F University, Yangling, Shaanxi, China (N 34°10′, E 108°10′, 526 m elevation). The experiment was laid-out in Randomized Complete Block Design with two replications. Each genotype was planted in 3 rows of 2 m length with row-to-row and plant-to-plant distance of 25 cm and 6.7 cm, respectively. All genotypes were sown under natural field conditions solely dependent on the soil moisture and the natural rainfall in season.

**Table 1:** Details of the genotypes used in the current study.

Code	Name	Origin	Region	Code	Name	Origin	Region
1	Luohan 2	Henan	HHWWR	31	Aifeng 3	Shaanxi	HHWWR
2	Shijiazhuang 8	Hebei	NWWR	32	Bainong 160	Henan	HHWWR
3	Jinmai 47	Shanxi	NWWR	33	Shaanhan 187	Shaanxi	HHWWR
4	Linhan51329	Shanxi	NWWR	34	Shijiazhuang 54	Hebei	NWWR
5	Shaan 229	Shaanxi	HHWWR	35	Luomai 21	Henan	HHWWR
6	Xiaoyan 6	Shaanxi	HHWWR	36	Lunxuan 061	Beijing	NWWR
7	Pubing 143	Shaanxi	HHWWR	37	Luo 9908	Henan	HHWWR
8	Zhonghan 110	Beijing	NWWR	38	Heng95Guan26	Hebei	NWWR
9	Liken 2	Shaanxi	HHWWR	39	Jinmai 33	Shanxi	NWWR
10	Changwu135	Shaanxi	HHWWR	40	Kedong 81	Beijing	NWWR
11	Linfen 10	Shanxi	NWWR	41	Shaanken 81	Shaanxi	HHWWR
12	Luohan 3	Henan	HHWWR	42	Han 6172	Hebi	NWWR
13	Linhan536	Shanxi	NWWR	43	Huaimai 21	Jiangsu	HHWWR
14	Jing 411	Beijing	NWWR	44	Yunong 982	Henan	HHWWR
15	Tongmai 3	Shaanxi	HHWWR	45	Xifeng 20	Gansu	HHWWR
16	Mianyang 11	Sichuan	SWWWR	46	Lunxuan 715	Beijing	NWWR
17	Xinyuan 958	Henan	HHWWR	47	Nongda 198	Beijing	NWWR
18	Linfen 10	Shanxi	NWWR	48	Fengkang 5	Beijing	NWWR
19	Taishan 5	Shandong	NWWR	49	Luohan 6	Henan	HHWWR
20	Jining 18	Shandong	NWWR	50	Jingwang 9	Beijing	NWWR
21	Xinmai 13	Henan	HHWWR	51	Jingdong 1	Beijing	NWWR
22	Youmai 2	Shandong	NWWR	52	Jinmai 21	Shanxi	NWWR
23	Xinmai 18	Henan	HHWWR	53	Jimai 23	Hebei	NWWR
24	Xinong 2000–7	Shaanxi	HHWWR	54	Jinan 18	Shandong	NWWR
25	Shaanmai 150	Shaanxi	HHWWR	55	Hanxuan 1	Shanxi	NWWR
26	Zhoumai 16	Henan	HHWWR	56	Lumai 1	Shandong	NWWR
27	Yuanfeng 139	Shaanxi	HHWWR	57	Wenmai 6	Henan	HHWWR
28	Fengchan 3	Shaanxi	HHWWR	58	Yunhan 618	Shanxi	NWWR
29	Xinong 979	Shaanxi	HHWWR	59	Hanxuan 10	Shanxi	NWWR
30	Zhongyu 8	Henan	HHWWR				

**Note:** HHWWR: Huang-huai Winter Wheat Region; NWWR: Northern Winter Wheat Region; SWWWR: Southwestern Winter Wheat Region.

### Identification and sequence analysis of *TaRca2-α*

DNA sequence of wheat Rubisco activase (*TaRca2-α*, accession No. LM992845) was used to search the homoeologous copies through BLAST against wheat chromosome sequence survey database (http://wheatgenome.org). The exon/intron distribution of the three copies was predicted using spidey tool in NCBI database (http://ncbi.nlm.nig.gov/spidey/). The predicted amino acid sequences of the three copies were fruther determined through ExPASy (http://web.expasy.org/translate/). The conserved domains were analyzed using Conserved Domain Search of NCBI database (http://www.ncbi.nlm.nih.gov/Structure/cdd/wrpsb.cgi).

### Primer designing for expression analysis of *TaRca2-α* by qRT-PCR

Multiple alignments of the 3′ untranslated regions (UTRs) at the C-terminal extension on the three copies of *TaRca2-α-4AL*, *TaRca2-α-4BS* and *TaRca2-α-4DS* were carried-out using ClustalW program in Bioedit 7.0 [19]. Primer pairs were hand-picked based on sequence polymorphisms at the 3′ ends of their forward and reverse sequences. General properties of primers picked were further checked using PrimerPREMIER version 5.0 (PREMIER Biosoft International). The genome-specificities of the primer pairs were tested with RT-PCR using RNA from three nulli-tetrasomic (NT) lines (N4AT4B, N4BT4D and N4DT4A) of Chinese Spring. Three reference genes (*TaActin*, *TaSand* and *TaCell*) were used for background standardization in wheat [20]. The details of the primers used are given in Table 2.

**Table 2:** Primer pairs used for qRT-PCR analysis in 59 bread wheat genotypes.

Name of primer	Primer sequence (5′ - 3′)	Usage
*TaRca2- α _AF*	GGTGTCTGCAAGGGTATCTTC	*TaRca2-α-4AL*
*TaRca2- α _AR*	TCGACTGTCATCTTTGGCTG	
*TaRca2- α _BF*	ACGCCGACCAACTTCCTT	*TaRca2-α-4BS*
*TaRca2- α _BR*	CAAGACCCTTCCACTTGTCC	
*TaRca2- α _DF*	GACGAGAAGAGGAACACC	*TaRca2-α-4DS*
*TaRca2- α _DR*	TGGCTGACGTACTCGTAT	
*TaActin_F*	TTGCTGACCGTATGAGCAAG	Reference gene *TaActin*
*TaActin_R*	ACCCTCCAATCCAGACACTG	
*TaSand_F*	TGCCTTGCCCATAAGAAATC	Reference gene *TaSand*
*TaSand_R*	GTGCGGACCAGTTGCTTTAT	
*TaCell_F*	GAGGAGGATGAGGTGGATGA	Reference gene *TaCell*
*TaCell_R*	CCTGGTACTTGCGGATGTCT	

### Total RNA isolation and cDNA synthesis

Fully expanded flag leaf samples of five randomly chosen plants from each of the 59 experimental genotypes were taken and pooled together, respectively at heading (Z55), anthesis (Z65) and grain-filling (Z73) stages. A three-step RNA extraction was carried out using modified hot phenol method [21, 22]. Initial extraction was carried out in 1 mL (80°C) 1:1 Phenol/Extraction buffer (0.1 M Tris-HCL, pH 8.0, 0.1 M LiCl, 1% (w/v) SDS and 10 mM EDTA). Afterwards, two phenol/chloroform/IAA (25:24:1) extractions were conducted. Genomic DNA contamination was removed with DNaseI (TAKARA, Dalian) treatment according to the manufacturer’s instruction. The first strand cDNA was synthesized from 10 μg of total RNA from each template with PrimScriptIII RT-PCR kit (TAKARA, Dalian) using oligo (dT)_18_ primer according to the manufacturer’s instructions. The cDNA samples were stored at -20°C for subsequent analysis.

### Expression analysis by qRT-PCR

cDNA sample from each genotype was replicated three times as per specifications of the SYBER Premix ExTaq Kit (Takara, Dalian), qRT-PCR were conducted using ABI7300 real time PCR system (Applied Bio Systems, USA). The reaction mixture was consisted of a total volume of 20μl including 10μl 2X SYMBER MIX, 0.3μl of each of the forward and reverse primer (0.6 μM), 1.5μl template cDNA (100 ng), ddH_2_O was added to get the final volume of 20μl. The qRT-PCR reaction was programmed as initial denaturation at 95°C for 20s, followed by 40 cycles at 95°C for 5s, 60°C for 30s. Relative expression of the target gene was calculated as under:

$$NE=\frac{{\left(E_{X}\right)}^{-Ct,X}}{{\left(E_{R}\right)}^{-Ct,R}}$$

Where *NE* is the relative expression of target gene, *E* is the primer efficiency, *Ct* value is collected where the fluorescence is above the thresh-hold value, *X* indicates values from the target gene, *R* indicates the geometric mean of values from the three reference genes [23, 24].

### Phenotypic evaluation

Net photosynthesis rate (Pn) was determined on fully expanded flag leaves of 5 randomly selected plants in each plot of each replication at heading (Z55), anthesis (Z65) and grain-filling stages(Z73), respectively, using portable photosynthesis system (LI6400XT, USA). The leaf chamber’s conditions were as reference CO_2_ concentration = 400 μmol mol^-1^, PPFD = 1800 μmol m^-2^ s^-1^, relative humidity = 50–70% and block temperature = 20°C. The measurements were taken between 9:00 and 11:00 am in sunny and windless conditions.

At maturity, 10 plants from each plot and each replication were randomly selected; the above-ground plant parts were harvested, dried and weighed for biomass plant^-1^ (g) using electronic balance. The same 10 plants were then threshed separately to record grain yield plant^-1^ (g).

### Data analysis

Analysis of variance (ANOVA) was conducted for the expressions of *TaRca2-α-4AL*, *TaRca2-α-4BS* and *TaRca2-α-4DS* at heading (Z55), anthesis (Z67) and grain-filling (Z73) stages. Separate analysis at heading (Z55), anthesis (Z67) and grain-filling (Z73) stages were also carried-out for Pn, whereas ANOVA for the final BMPP and GYPP was conducted after harvest. Hierarchical cluster analyses were performed to classify the 59 bread wheat genotypes on the basis of average expression of *TaRca2-α-4AL*, *TaRca2-α-4BS* and *TaRca2-α-4DS* across the three growth stages. Correlation coefficients between the expressions of the three copies at the three stages with the measured traits at the respective stages were determined using Pearson Product Moment Correlation test. All statistical analyses were carried-out using SPSS statistical software version 19.0 (IBM SPSS Statistics, USA).

## Results

### Characterization of the *TaRca2-α*gene in wheat

BLAST search using DNA sequence of *TaRca2-α* (accession No. LM992845) against wheat chromosome sequence survey database (http://wheatgenome.org) revealed that there were three homoeologous copies of *TaRca2-α* located on long arm of chromosome 4A, and short arms of chromosome 4B and 4D, respectively, and designated as *TaRca2-α-4AL*, *TaRca2-α-4BS* and *TaRca2-α-4DS*. The predicted size of *TaRca2-α-4AL*, *TaRca2-α-4BS* and *TaRca2-α-4DS* was 1719 bp, 1749 bp, and 1735 bp in length, respectively. Analysis using spidey tool in NCBI database (http://ncbi.nlm.nih.gov/spidey/) predicted five exons and four introns for each copy, and the coding sequences (CDS) of the three copies were 1193 bp (*TaRca2-α-4AL*), 1195 bp (*TaRca2-α-4BS*) and 1195 bp (*TaRca2-4DS*), respectively. Multiple sequence alignment of CDS of the three copies revealed 26 nucleotide difference between *TaRca2-α-4AL* and *TaRca2-α-4BS*, 29 nucleotide difference between *TaRca2-α-4BS* and *TaRca2-α-4DS*, and 19 nucleotide difference between *TaRca2-α-4AL* and *TaRca2-α-4DS* (S1 Fig). The three copies encoded 397, 397 and 396 amino acids for *TaRca2-α-4AL*, *TaRca2-α-4BS* and *TaRca2-α-4DS*, respectively (S2 Fig). High similarities were observed in the conserved protein (AAA) domains of the three copies (S2 Fig).

### Expression of *TaRca2-α* in 59 bread wheat genotypes

The specificity of individual primer pairs for the three copies of *TaRca2-α* was tested in three nulli-tetrasomic lines (N4ATB, N4BT4D and N4DT4A) of Chinese Spring. The single RT-PCR product for *TaRca2-α-4AL*, *TaRca2-α-4BS* and *TaRca2-α-4DS* was 233, 151 and 164 bp, respectively. The 4AL-specific primer pair amplified a single PCR product from N4BT4D and N4DT4A, but not from N4ATB. The amplicon produced by the 4BS-specific primer pair was absent in N4BT4D, but present in N4AT4D and N4DT4A. The 4DS-specific primer set generated a single PCR product from N4AT4B and N4BT4D, but not from N4DT4A (S3 Fig).

Expression analysis by qRT-PCR showed that the three copies of *TaRca2-α* were highly expressed at grain-filling stage (0.68) than at heading (0.45) and anthesis (0.54) stages (Table 3). Significantly different expression patterns at the three growth stages and among those genotypes were observed for *TaRca2-α-4AL* with a range of 0.18 to 0.83 at heading, 0.32 to 1.18 at anthesis and 0.45 to 1.22 at grain-filling stages (*P*<0.05) (Table 3). Similar results were observed for *TaRca2-α-4BS* expression, which ranged between 0.25 and 1.07 at heading, 0.30 to 1.26 at anthesis and 0.49 to 1.35 at grain-filling stages (Table 3). No significant (*P*<0.05) differences for *TaRca2-α-4DS* expression were found between heading and anthesis stages, whereas it was significantly higher at grain-filling stage with a range of 0.41 to 1.29 (Table 3). Overall, *TaRca2-α-4BS* showed higher expression at the three growth stages than *TaRca2-α-AL* and *TaRca2-α-4DS* (Table 3). The expressions of three copies of *TaRca2-α* at the three growth stages were significantly (*P*<0.01) and positively correlated with each other, with the highest correlations at grain-filling stage. The correlation coefficient (r) between *TaRca2-α-4AL* and *TaRca2-α-4BS*, *TaRca2-α-4AL* and *TaRca2-α-4DS*, *TaRca2-α-4BS* and *TaRca2-α-4DS* at grain-filling stage was 0.916, 0.928, and 0.954, respectively (S1 Table).

**Table 3:** Expression of the three copies of *TaRca2-α* at three growth stages in flag leaves of 59 bread wheat genotypes.

*TaRca2- α g*ene	Item	Heading (Z55)	Anthesis (Z67)	Grain-filling (Z73)
*TaRca2- α -4AL*	Mean	0.41±0.018 C	0.57±0.020 B	0.67±0.021 A
	Range	0.18–0.83	0.32–1.18	0.45–1.22
*TaRca2- α -4BS*	Mean	0.54±0.024 C	0.63±0.029 B	0.76±0.029 A
	Range	0.25–1.07	0.30–1.26	0.49–1.35
*TaRca2- α -4DS*	Mean	0.38±0.019 B	0.42±0.020 B	0.61±0.022 A
	Range	0.15–0.94	0.11–0.84	0.41–1.29
Overall	Mean	0.45±0.019 C	0.54±0.0.21 B	0.68±0.023 A
	Range	0.22–0.93	0.27–1.02	0.45–1.29

Data are shown as the mean ± SE (standard error) of all genotypes; uppercase letters represent differences significant among the three growth stages (*P*≤0.01).

**Fig 1:**
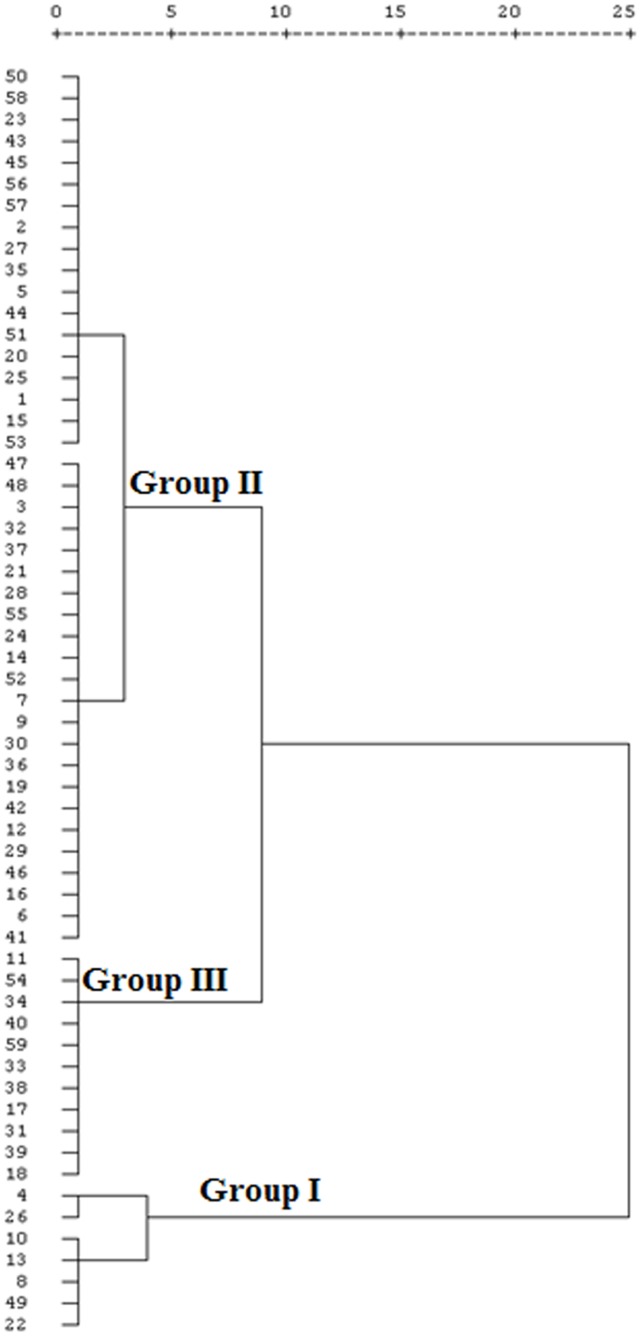
Cluster analysis of 59 bread wheat genotypes based on averaged expressions of three copies of *TaRca2- α*. Group I: high expression, Group II: intermediate expression; Group III: low expression. The numbers on Y axis represent the codes of 59 wheat genotypes as in Table 1. X axis represents the squared Euclidean distance.

Based on the relative expression of the three homoeologous copies of *TaRca2-α* at the three growth stages, the 59 wheat genotypes could be clustered into three groups (Fig 1). The group I was comprised of 7 genotypes with high expression, Group II contained 41 genotypes with intermediate expression, whereas group III was consisted of 11 genotypes with low expression.

Significant differences among the three groups of bread wheat genotypes were observed on the expressions of the three copies of *TaRca2-α* at heading, anthesis and grain-filling stages (Table 4). For all the three copies of *TaRca2-α*, the group I genotypes always revealed the highest mean expression, and the group III genotypes showed the lowest, while the group II genotypes expressed the intermediate mean expression (Table 4). At each growth stage, the group I genotypes showed significantly higher expressions (0.67, 0.67 and 0.81 at heading, anthesis and grain-filling stages, respectively), whereas the group III genotypes observed the lowest expressions (0.22, 0.21 and 0.43 at heading, anthesis and grain-filling stages respectively).

**Table 4:** Expression of the three copies of *TaRca2-α* at heading (Z755), anthesis (Z67) and grain-filling (Z73) stages in the three groups of 59 bread wheat genotypes.

*TaRca2-α g*ene	Growth Stage	Items	Group I	Group II	Group III
*TaRca2- α -4AL*	Heading (Z55)	Mean	0.66±0.042 A	0.42±0.011 B	0.22±0.006 C
		Range	0.60–0.83	0.30–0.53	0.18–0.24
	Anthesis (Z67)	Mean	0.86±0.058 A	0.57±0.012 B	0.38±0.006 C
		Range	0.75–1.18	0.45–0.73	0.32–0.39
	Grain-filling (Z73)	Mean	1.02±0.068 A	0.66±0.009 B	0.51±0.008 C
		Range	0.80–1.22	0.57–0.74	0.45–0.53
*TaRca2- α -4BS*	Heading (Z55)	Mean	0.97±0.026 A	0.53±0.007 B	0.31±0.014 C
		Range	0.90–1.07	0.49–0.60	0.25–0.40
	Anthesis (Z67)	Mean	1.18±0.028 A	0.59±0.005 B	0.42±0.019 C
		Range	1.09–1.26	0.53–0.71	0.30–0.48
	Grain-filling (Z73)	Mean	1.26±0.027 A	0.74±0.014 B	0.50±0.003 C
		Range	1.15–1.35	0.60–0.90	0.49–0.53
*TaRca2- α -4DS*	Heading (Z55)	Mean	0.67±0.067 A	0.37±0.011 B	0.22±0.008 C
		Range	0.55–0.94	0.24–0.49	0.15–0.24
	Anthesis (Z67)	Mean	0.67±0.044 A	0.43±0.014 B	0.21±0.020 C
		Range	0.59–0.84	0.29–0.54	0.11–0.30
	Grain-filling (Z73)	Mean	0.98±0.063 A	0.59±0.015 B	0.43±0.004 C
		Range	0.81–1.29	0.45–0.77	0.49–0.53

Data are shown as the mean ± SE (standard error) of the genotypes in each group; Group I: high expression; Group II: intermediate expression; Group III: low expression. Uppercase letters represent differences significant among the three groups (*P*≤0.01).

**Fig 2:**
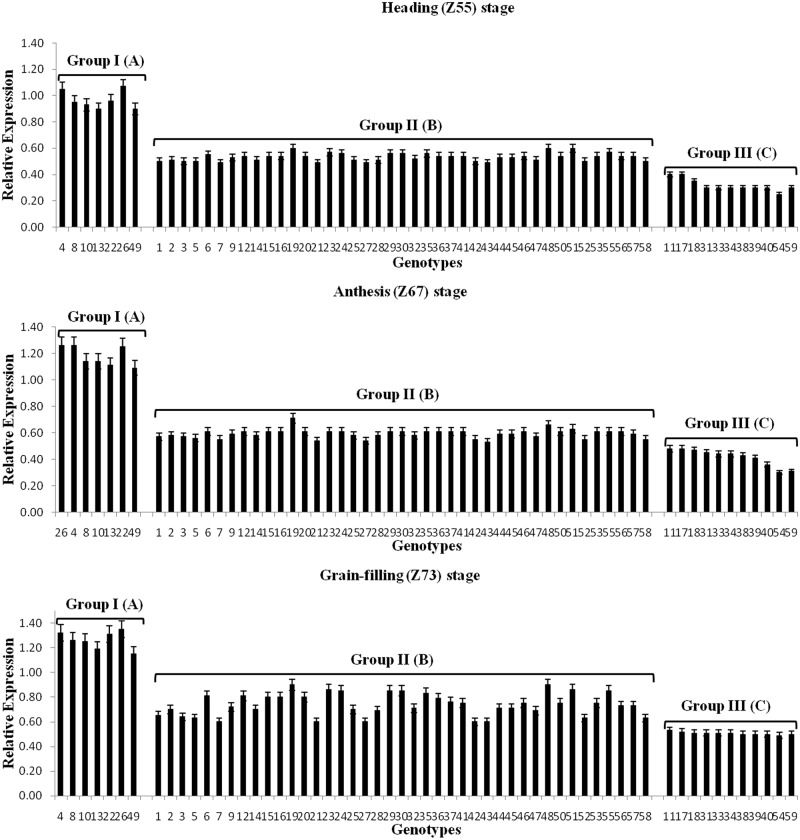
*TaRca2-α-4BS* expression in flag leaves of the three groups of 59 bread wheat genotypes at Heading (Z55), Anthesis (Z67) and Grain-filling (Z73) stages. Group I: high expression; Group II: intermediate expression; Group III: low expression. Uppercase letter represent significant differences among the three groups (*P*≤0.01). The numbers on X-axis correspond to the codes of individual genotypes in Table 1.

Significant differences were also observed among individual genotypes for the expressions of the three copies of *TaRca2-α* at the three growth stages (Fig 2, S2 Table). In general, the individual genotypes showed similar expression trends for the three copies at the three growth stages. For instance, the 7 genotypes (Zhoumai 16, Linhan 51329, Youmai 2, Zhonghan 110, Changwu 135, Linhan 536 and Luohan 6) in group I expressed *TaRca2-α-4BS* at higher level at all three stages (Fig 2, S2 Table), while the genotypes Jinan 18 and Aifeng 3 in group III expressed the lowest at all three stages.

### Associations between *TaRca2-α* expression and Pn

**Fig 3:**
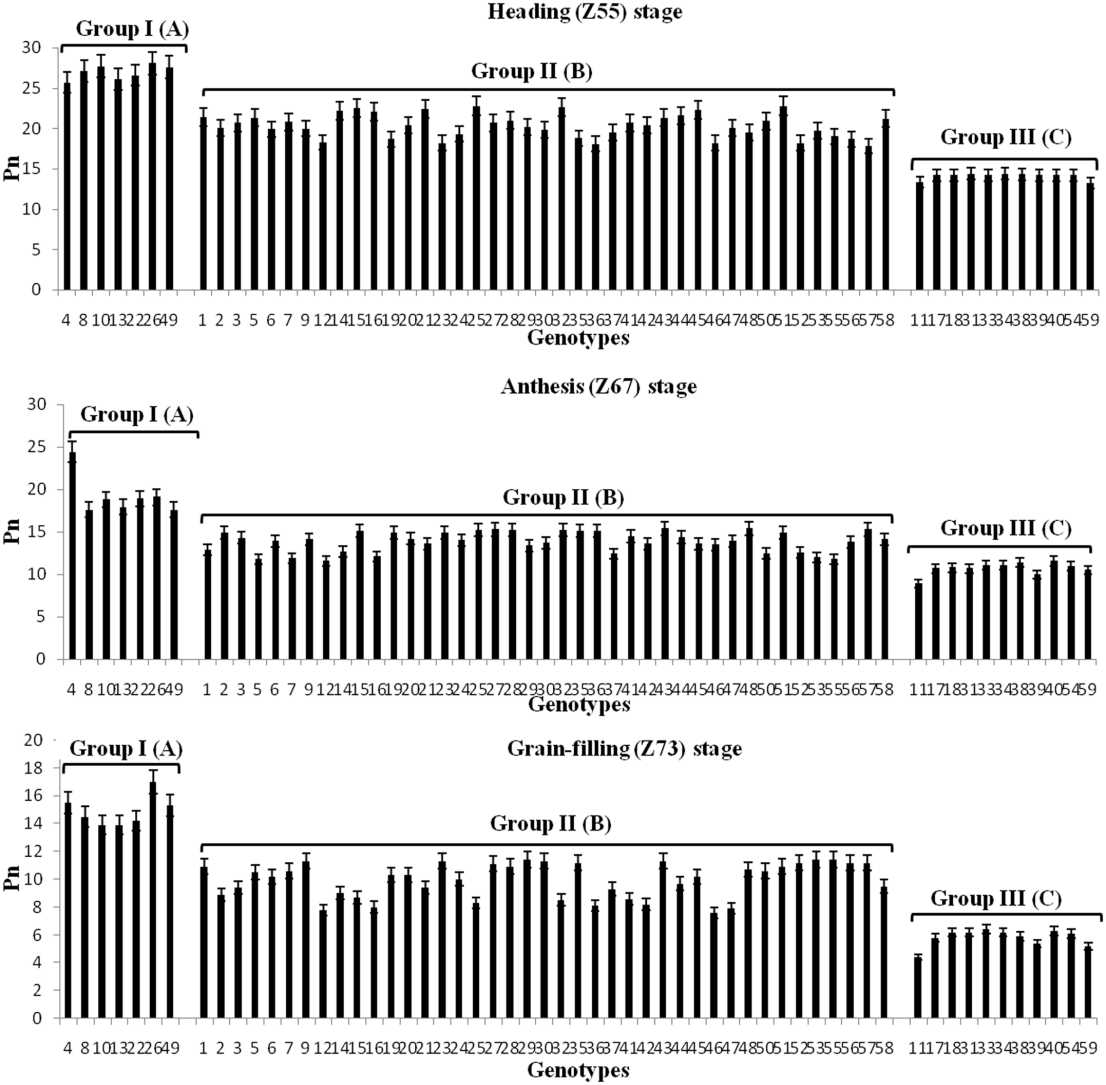
Pn (μmol m-2 s-1) of 59 bread wheat genotypes in the three groups at heading (Z55), anthesis (Z67) and grain-filling (Z73) stages. Group I: high expression; Group II: intermediate expression; Group III: low expression. Uppercase letter represent significant differences (*P*≤0.01) among the three groups. The numbers on X-axis correspond to the codes of individual genotypes in Table 1.

Significant differences for Pn among the wheat genotypes of the three groups were observed at all three growth stages, with the highest (20.4 μmol m^-2^ s^-1^) at heading and the lowest (10.2 μmol m^-2^ s^-1^) at grain-filling stage (Table 5; Fig 3). In general, the genotypes with high *TaRca2-α* expression at the three growth stages also showed higher Pn at the three growth stages. For instance, at heading stage, the highest Pn was recorded in all seven genotypes in group I (25.7 to 28.1 μmol m^-2^ s^-1^), whereas the lowest Pn was observed in Hanxuan 10 (13.2 μmol m^-2^ s^-1^), Linfen 10 (13.5 μmol m^-2^ s^-1^) and Jinan 18 (14.3 μmol m^-2^ s^-1^) from group III (S3 Table).

**Table 5:** Mean Pn (μmol m^-2^ s^-1^) of the three groups of 59 bread wheat genotypes at heading (Z55), anthesis (Z67) and heading (Z73) stages.

Pn	Item	Group I	Group II	Group III	Average
Pn-Heading (Z55)	Mean	26.9±0.336 A	20.4±0.229 B	14.0±0.120 C	20.4±0.454 A
	Range	25.7–28.1	17.8–22.8	13.2–14.5	13.2–28.1
Pn-Anthesis (Z67)	Mean	19.2±0.901 A	13.8±0.186 B	10.8±0.223 C	14.6±0.342 B
	Range	17.6–24.4	11.6–15.4	8.9–11.6	8.9–24.4
Pn-Grain-filling (Z73)	Mean	14.9±0.425 A	9.9±0.195 B	5.8±0.183 C	10.2±0.354 C
	Range	13.9–17.0	7.6–11.4	4.4–6.4	4.4–17

Data are shown as the mean ± SE (standard error) of the genotypes in each group; Group I: High expression; Group II: medium expression; Group III: low expression. Uppercase letters represent significant differences between the three groups of (*P*<0.01).

**Fig 4:**
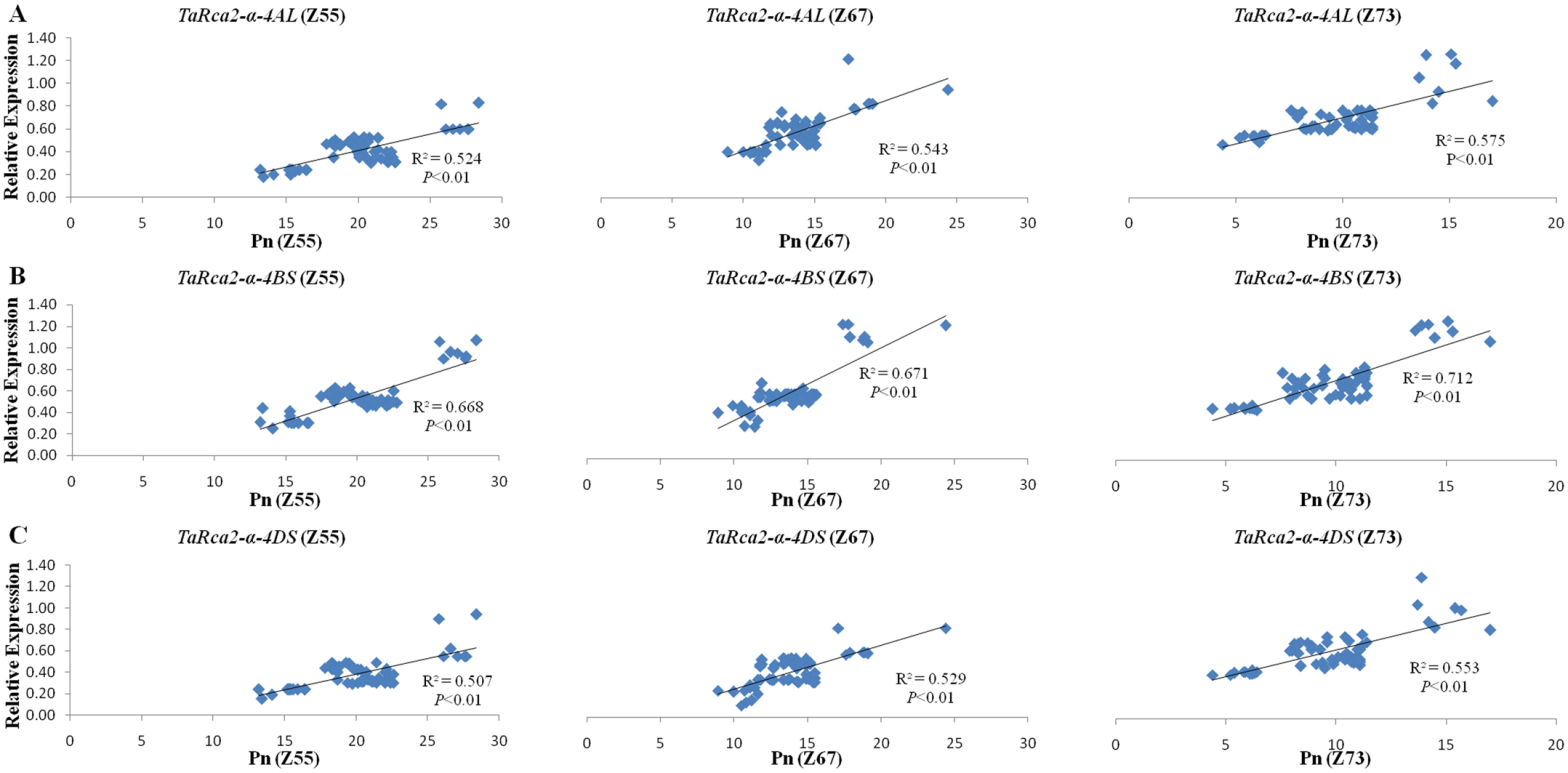
Regression analysis between the expressions of the three copies of *TaRca2-α*with Pn (μmol m^-2^ s^-1^) at heading, anthesis and grain filling stages. A: *TaRca2-α-4AL*; B: *TaRca2-α-4BS*; C: *TaRca2-α-4DS*. Pn (Z55), Pn (Z67), Pn (Z73), indicate the Pn at heading (Z55), anthesis (Z67) and grain filling (Z73) stages, respectively.

Regression analysis showed that expressions of the three copies of *TaRca2-α* at heading, anthesis and grain-filling stages were significantly and positively (*P*<0.01) associated with Pn at all the corresponding growth stages (Fig 4). The expressions of *TaRca2-α-4BS* were more strongly correlated with Pn than that of *TaRca2-α-4AL* and *TaRca2-α-4DS*, with regression coefficients of 0.678, 0.671 and 0.712 at heading, anthesis and grain-filling stages, respectively. The expressions of *TaRca2-α-4AL*, *TaRca2-α-4BS* and *TaRca2-α-4DS* were highly correlated with Pn at grain-filling stage, with regression coefficients of 0.575, 0.712 and 0.553, respectively, than at heading and grain-filling stages.

### Correlations between *TaRca2-α* expression with BMPP and GYPP

**Fig 5:**
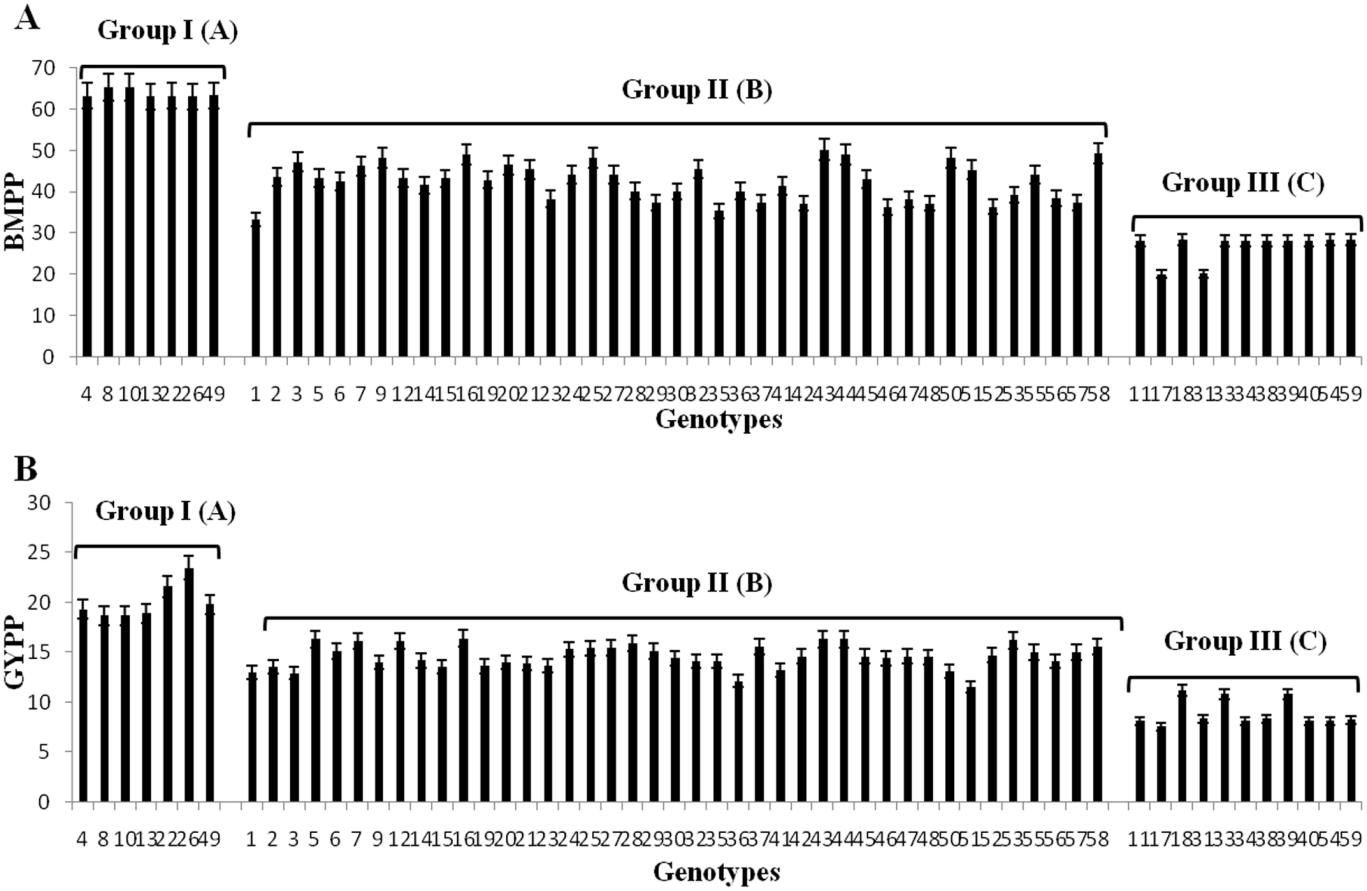
BMPP and GYPP of bread wheat genotypes in three groups. Group I: high expression; Group II: intermediate expression; Group III: low expression. Uppercase letters represent significant differences (P<0.01) level. The numbers on X-axis correspond to the codes of individual genotypes in Table 1.

Significant differences were observed among the three groups of bread wheat genotypes for biomass plant^-1^ (BMPP) and grain yield plant^-1^ (GYPP) (Table 6, Fig 5). Generally, genotypes showing high *TaRca2-α* expression produced higher BMPP and GYPP. Group I genotypes with high expression produced the highest mean BMPP of 63.8 g plant^-1^and GYPP of 20.0 g plant^-1^, group II genotypes with intermediate expression showed medium mean BMPP of 42.3 g plant^-1^ and GYPP of 14.5 g plant^-1^, whereas group III genotypes with low expression produced the lowest average BMPP of 26.7 g plant^-1^ and GYPP of 8.8 g plant^-1^. The maximum BMPP was produced by Changwu 135 (65.4 g plant^-1^) and Zhonghan 110 (65.3 g plant^-1^) in group I, whereas the lowest BMPP was recorded in Xinyuan 958 (20.1g plant^-1^) and Aifeng 3 (20.2 g plant^-1^) in group III. The highest GYPP was recorded in genotype Zhoumai 16 (23.5 g plant^-1^) of group I, while the lowest GYPP was in genotype Xinyuan 958 (7.5g plant^-1^) of group III (S3 Table).

**Table 6:** Mean BMPP (g plant^-1^) and GYPP (g plant^-1^) of three groups of 59 bread wheat genotypes.

Trait	Item	Group I	Group II	Group II
BMPP	Mean	63.8±0.401 A	42.3±0.702 B	26.7±0.987 C
	Minimum	63.1	33.3	20.1
	Maximum	65.4	50.2	28.4
GYPP	Mean	20.0±0.688 A	14.5±0.187 B	8.8±0.407 C
	Minimum	18.7	11.5	7.5
	Maximum	23.5	16.4	11.2

Data are shown as the mean ± SE (standard error) of the genotypes in each group; Group I: high expression; Group II: intermediate expression; Group III: low expression. Uppercase letters represent significant difference among the three groups (*P*<0.01).

**Fig 6:**
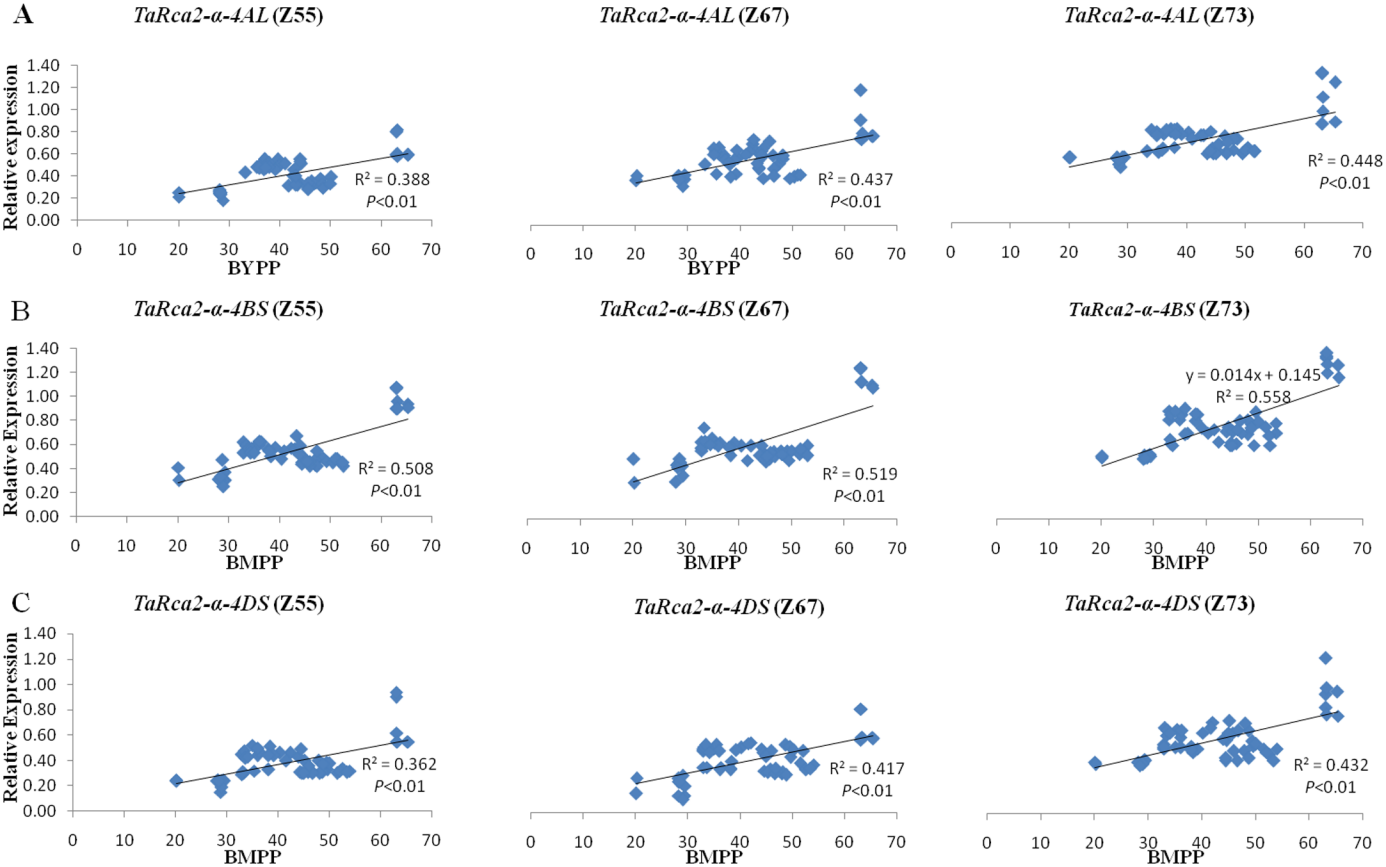
Regression analysis between the expressions of the three copies of *TaRca2-α* at heading, anthesis and grain filling stages, with BMPP. A: *TaRca2-α-4AL*; B: *TaRca2-α-4BS*; C: *TaRca2-α-4DS*. Z55, Z67 and Z73, indicate the expression measured at heading (Z55), anthesis (Z67) and grain filling (Z73) stages, respectively.

**Fig 7:**
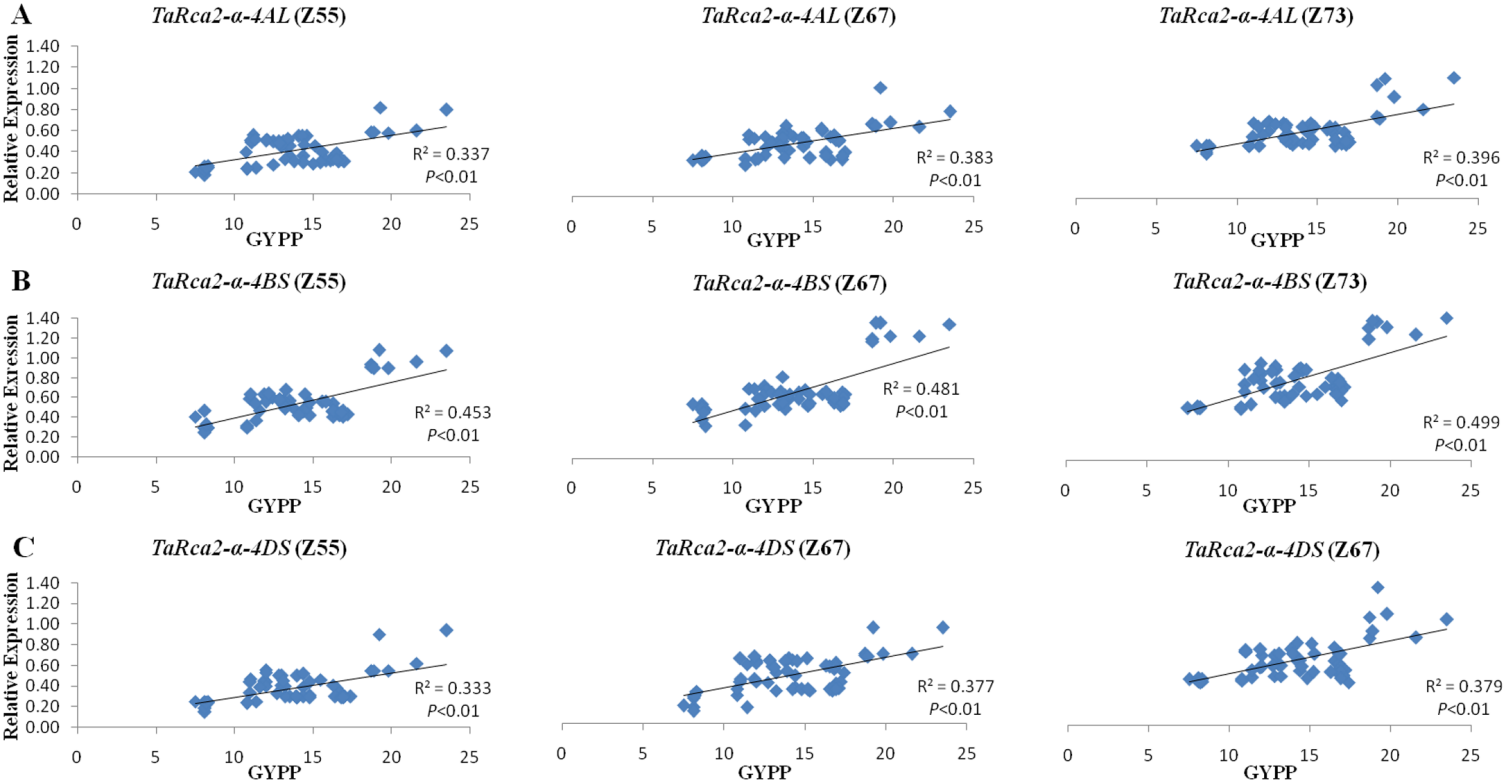
Regression analysis between the expressions of the three copies of *TaRca2-α* at heading, anthesis and grain filling stages, with GYPP. A: *TaRca2-α-4AL*; B: *TaRca2-α-4BS*; C: *TaRca2-α-4DS*. Z55, Z67 and Z73, indicate the expression measured at heading (Z55), anthesis (Z67) and grain filling (Z73) stages, respectively.

Significant and positive associations (*P*<0.01) were observed between the expression of all the three copies at the three growth stages with BMPP and GYPP as revealed by regression analysis (Figs 6 and 7). The expressions of all three copies of *TaRca2-α* at grain-filling were more strongly correlated with BMPP and GYPP with regression coefficients of 0.448, 0.558 and 0.432 for BMPP and of 0.396, 0.499 and 0.379 for GYPP with the three copies of *TaRca2-α*, respectively than that at heading and anthesis stages (Figs 6 and 7). In comparison with *TaRca2-α-4AL and TaRca2-α-4DS*, *TaRca2-α-4BS* expression showed stronger positive correlations with BMPP and GYPP at all three growth stages, with regression coefficient of 0.508, 0.519 and 0.588 for BMPP and of 0.453, 0.481 and 0.499 for GYPP with its expression at heading, anthesis and grain-filling stages, respectively (Figs 6 and 7).

## Discussion

### Expression patterns of *TaRca2-α* in wheat

In bread wheat, *TaRca2-α* is produced as a result of splicing event at the end of exon-5 of *TaRca2-β* [8]. The *α* and *β* isoforms are capable of supporting photosynthesis with increased expression and contribution of *α* isoform to plant productivity under different growth conditions [25, 26, 27]. It suggests the important role of *α* isoform of *Rca* as a molecular chaperon in protecting other functional proteins from damage [16]. The present research was conducted to study the expression of *TaRca2-α* at heading (Z55), anthesis (Z67) and grain-filling (Z73) stages under natural field conditions where macro/micro climate is not steady and plants face certain limiting factors. Overall, our results confirmed the higher variation in the expression of *TaRca2-α* in the panel of 59 bread wheat genotypes grown under field conditions (Table 3). Most of the previous studies were conducted under controlled growth conditions, whereas little information is available regarding investigation of *Rca* genes under natural conditions especially in wheat. However, results of the current study are in agreement with earlier studies in other crops [16, 17] in which the investigators reported higher expression of *α* isoform with positive effects on plant phenotypes under controlled as well as natural field conditions. Being allopolyploid, wheat has three homoeologous copies of a gene in general and study of the three copies helps to know the contribution of a specific copy [18]. In the current study, the three copies of *TaRca2-α* were identified in bread wheat, and then genome-specific primers were designed to investigate their expression levels in flag leaves at the three growth stages under field condition. The expressions of all three copies of *TaRca2-α* were higher at grain-filling (Z73) stage than at heading (Z55) and anthesis (Z67) stages, as similar expression patterns for *Rca-α* were also reported in other crops [28, 29], which suggests positive contribution of expression at the grain-filling stage to the plant productivity and grain yield under prevailing environmental conditions. *TaRca2-α-4BS* was more highly expressed than *TaRca2-α-4AL* and *TaRca2-α-4DS*, which were in consistency with those by Carmo-Silva et al. [8], which suggests that *TaRca2-α-4BS* might be more important and may contribute to enhanced plant performance. In a research on wheat, Edae et al [30] studied the association of homoeologous copies of *DREB1*, *ERA1* and *1-FEH*, and reported relatively strong association of one copy with traits compared to other copies indicating the important role of a specific homoeologue in controlling agronomic traits.

Diversity in gene expression is one of the mechanisms underlying phenotypic variation among genotypes [17] and aids in identification of genotypes with better traits [16, 20, 31]. In the present work, significant variations on the expression levels of the three copies of *TaRca2-α* at the three growth stages were also observed among the bread wheat genotypes in the panel, therefore the genotypes were clustered into groups based on the relative expression of *TaRca2-α* to facilitate the analysis for determining whether those variations were associated with their photosynthetic capability and performance regarding biomass and grain yield. As shown in the cluster results, 41 bread wheat genotypes (69.5%) were with intermediate expressions of *TaRca2-α*, and only 7 genotypes with higher expressions, similar results were found in our previous work with *TaER* genes in bread wheat [20].

### Association of *TaRca2-α* expression with Pn

Rubisco activase is the key enzyme for net CO_2_ assimilation in C_3_ crops through activating Rubisco [9, 32, 33] and the *α* isoform of *Rca* plays an important role in maintaining Rubisco’s activity and hence plant efficiency under various conditions [15, 16]. In the present study, we observed that the genotypes showing high *TaRca2-α* expression levels at the three growth stages also expressed high Pn values at the corresponding stages, and the expression levels of the three copies of *TaRca2-α* at the three stages were all significantly and positively correlated with Pn at the corresponding stages with comparatively stronger association for *TaRca2-α-4BS* and at grain-filling stage. This possibly suggested the great contribution of *TaRca2-α* to Pn, with a more significant contribution by *TaRca2-α-4BS*. These are in agreement with the previous findings in soybean, maize and other crops [16, 20, 28, 29, 34]. Grain-filling is a crucial stage for achieving optimum grain yield, the relatively stronger association of the expression levels of *TaRca2-α* with Pn at this stage suggested that higher expression may contribute more to grain yield, although Pn reduced greatly at this stage as shown in other studies [5, 17]. These results support the hypothesis that *TaRca2-α* contributed to photosynthesis substantially, detection of its expression levels could be utilized as a selection tool for the improvement of photosynthetic efficiency in bread wheat The genomic variations resulting in this expression difference should be further clarified for execution in marker assisted selection.

### Association of *TaRca2-α* expression with BMPP and GYPP

Studies in wheat and other C_3_ crops have shown that there exists a positive association between Pn and biomass [35] and grain yield [5], improvement in plant biomass can translate in reasonable gains in crop yield. A significant and positive correlation between Pn at heading, anthesis and grain-filling stages with BMPP and GYPP (*P*<0.01) were also observed in this study, which was more significant (r = 0.647 for BMPP and r = 0.511 for GYPP) at the grain-filling stage (Table 7). This indicates that there is a potential for an increase in grain yield through improvement in photosynthetic efficiency and biomass production. In wheat, Rubisco can potentially enhance CO_2_ assimilation resulting in biomass gain, the endogenous levels of *Rca* expression can be of importance to plant photosynthesis and biomass production [27, 28]. Investigation in this study revealed that, the expression levels of the three copies of *TaRca2-α* at the three growth stages were strongly and positively correlated with BMPP and GYPP with stronger effect of *TaRca2-α-4BS*, and also at grain-filling stage. In general, the genotypes showing high expression also produced higher BMPP and GYPP. These results are in concurrence with those reported in other crops under variable environmental conditions [15, 16, 17, 36, 37]. This suggests that regulation of *TaRca-α* expression may efficiently improve BMPP and GYPP in wheat and the variations in the expression levels of *TaRca-α* may be utilized for selection of biomass and grain yield among wheat genotypes under natural field conditions.

**Table 7:** Correlation coefficients between Pn at heading (Z55), anthesis (Z67) and grain-filling (Z73) stages with BMPP (g plant^-1^) and GYPP (g plant^-1^) in 59 bread wheat genotypes.

	**Pn-Heading (Z55)**	**Pn-Anthesis (Z67)**	**Pn-Grain-filling (Z73)**	**BMPP**	**GYPP**
**Pn-Heading (Z55)**	1				
**Pn-Anthesis (Z67)**	0.575**	1			
**Pn-Grain-filling (Z73)**	0.594**	0.611**	1		
**BMPP**	0.558**	0.574**	0.647**	1	
**GYPP**	0.476**	0.486**	0.511**	0.455**	1

**. Correlation is significant at the 0.01 level

Pn: net photosynthesis rate (μmol m^-2^ s^-1^); BMPP: biomass plant^-1^; GYPP: grain yield plant^-1^

The associations between the expression levels of *TaRca2-α* copies with Pn, BMPP and GYPP were studied in a panel of 59 bread wheat genotypes under field conditions. *TaRca2-α-4BS* was highly expressed as compared to *TaRc2-α-4AL* and *TaRca2-4DS*. The expression of the three copies of *TaRca2-α* was more profound at grain-filling (Z73) stage than at heading (Z55) and anthesis (Z67) stages, and were significantly and positively correlated with Pn, BMPP, and GYPP, which were stronger at grain-filling stage than at heading and anthesis stages, with comparatively stronger association with Pn. These results suggested that the expression of *TaRca2-α* contribute greatly to Pn, BMPP and GYPP, and regulation of *TaRca-α* expression may efficiently improve Pn, BMPP and GYPP in wheat, and the variations detected in TaRca2-α expression levels with special emphasis on *TaRca2-α-4BS* may be utilized for selection in improving photosynthetic efficiency and grain yield of bread wheat.

## Supporting Information

S1 Fig CDS of *TaRca2-α-4AL*, *TaRca2-α-4BS and TaRca2-α-4DS*. (DOCX)

S2 Fig Amino acids’ sequences of *TaRca2-α-4AL*, *TaRca2-α-4BS* and *TaRca2-α-4DS*. (DOCX)

S3 Fig Amplicons generated by genome-specific primers (*TaRca2-α-4AL*, *TaRca2-α-4BS and TaRca2-α-4DS*) in nulli-tetrasomic (NT) lines of Chinese Spring. (DOCX)

S1 Table Correlation coefficients between *TaRca2-α-4AL*, *TaRca2-α-4BS and TaRca2-α-4DS* in 59 bread wheat genotypes at heading (Z55), anthesis (Z67) and grain-filling (Z73) stages. (DOCX)

S2 Table Expression of *TaRca2-α-4AL*, *TaRca2-α-4BS and TaRca2-α-4DS* in 59 bread wheat genotypes at heading (Z55), anthesis (Z67) and grain-filling (Z73) stages. (XLSX)

S3 Table Pn at heading (Z55), anthesis (Z67) and grain-filling (Z73); and BMPP and GYPP in 59 bread wheat genotypes. (XLSX)

## References

[1] Reynolds M, Bonnett D, Chapman SC, Furbank RT, Manes Y, Mather DE, et al Raising yield potential of wheat. I. Overview of a consortium approach and breeding strategies. J Exp Bot. 2011; 62(2): 439–452. 10.1093/jxb/erq311 20952629

[2] FAO. 2015. http://faostat3.fao.org/home/E.

[3] RE Evenson, Gollin D. Assessing the impact of the green revolution, 1960 to 2000. Science. 2003; 300(5620): 758–762. 12730592 10.1126/science.1078710

[4] Rosegrant MW, Agcaoili M. Global food demand, supply, and price prospects to 2010. Washington, DC: International Food Policy Research Institute 2010.

[5] Fischer RA, Ress D, Sayre KD, Lu ZM, Condon AG, Saavedra AL. Wheat yield progress associated with higher stomatal conductance and photosynthetic rate, and cooler canopies. Crop Sci. 1998; 38: 1467–1475.

[6] Raven JA. Rubisco: still the most abundant protein on Earth? New Phytologist. 2013; 198, 1–3. 10.1111/nph.12197 23432200

[7] McNevin D, von Caemmerer S, Farquhar GD. Determining Rubisco activation kinetics and other rate and equilibrium constants by simultaneous multiple non-linear regression of a kinetic model. J Exp Bot. 2006; 57(14): 3883–3900. 17046981 10.1093/jxb/erl156

[8] Carmo-Silva E, Scales JC, Madjwick PJ, Parry MA. Optimizing Rubisco and its regulation for greater resource use efficiency. Plant Cell Environ. 2014; 38(9): 1817–32. 10.1111/pce.12425 25123951

[9] Portis AR Jr. Rubisco activase: Rubisco’s catalytic chaperone. Photosynth Res. 2003; 75(1):11–27. 16245090 10.1023/A:1022458108678

[10] Spreitzer RJ, Salvucci ME. Rubisco: Structure, regulatory interactions, and possibilities for a better enzyme. Annu Rev Plant Biol. 2002; 53: 449–475. 10.1146/annurev.arplant.53.100301.135233 12221984

[11] Neuwald AF, Aravind L, Spouge JL, Koonin EV. AAA+: a class of chaperone-like ATPases associated with the assembly, operation and disassembly of protein complexes. Genome Res. 1999; 9(1): 27–43. 9927482

[12] Portis AR Jr. The regulation of Rubisco by Rubisco activase. J Exp Bot. 1995; 46 (Special Issue):1285–1291. 10.1093/jxb/46.special_issue.1285

[13] Salvucci MM, Ogren WL. The mechanism of Rubisco activase: insights from studies of the properties and structure of the enzyme. Photosynth Res. 1996; 47(1)1–11. 10.1007/BF00017748 24301702

[14] Shen JB, Orozco EM Jr, Ogren WL. Expression of the isoforms of spinach ribulose 1,5-bisphosphate carboxylase acitvase and essentially of the conserved lysine in the consensus nucleotide-binding domain. J Biol Chem. 1991; 266(14): 8963–8968. 1827441

[15] Wang D, Li XF, Zhou ZJ, Feng XP, Yang WJ, Jiang DA. Two Rubisco activase isoforms may play different roles in photosynthetic heat acclimation in the rice plant. Physiol Plant. 2010; 139(1):55–67. 10.1111/j.1399-3054.2009.01344.x 20059735

[16] Chao M, Yin Z, Hao D, Zhang J, Song H, Ning A et al Variation in Rubisco activase (RCAα) gene promoters and expression soybean [Glycine max (L) Merr.]. J Exp Bot 2013; 65(1): 47–59. 10.1093/jxb/ert346 24170743 PMC3883283

[17] Yin Z, Zhenliang Z, Dexiang D, Maoni C, Qingsong G, Yijun W et al Characterization of Rubisco Activase Genes in Maize: An α-Isoform Gene Functions alongside a β-Isofrom Gene. Plant Physiol. 2014; 164(4): 2096–2106. 10.1104/pp.113.230854 24510763 PMC3982765

[18] Huang X-Q, Anita B-B. Development of genome-specific primers for homoeologous genes in allopolyploid species: the waxy and starch synthase II genes in allohexaploid wheat (Triticum aestivum L.) as example. BMC Research Notes. 2010; 3:140 10.1186/1756-0500-3-140 20497560 PMC2890506

[19] Hall TA. BioEdit: a user-friendly biological sequence alignment editor and analysis program for Windows 95/98/NT. Nucleic Acids Symposium Series. 1999; 41: 95–98.

[20] Zheng J, Yang Z, Madgwick PJ, Carmo-Silva E, Parry MAJ, Hu Y-G. TaER Expression is Associated with Transpiration Efficiency Traits and Yield in Bread Wheat. PLoS ONE. 2015; 10(6): e0128415 10.1371/journal.pone.0128415 26047019 PMC4457575

[21] Shinmachi F, Buchner P, Stroud JL, Parmar S, Zhao FJ, McGrath SP et al Influence of sulfur deficiency on the expression of specific sulfate transporters and the distribution of sulfur, selenium and molybdenum in wheat. Plant Physiol. 2010; 153(1): 327–336. 10.1104/pp.110.153759 20219830 PMC2862427

[22] Verwoerd TC, Dekker BM, Hoekema A. A small-scale procedure for the rapid isolation of plant RNAs. Nucleic Acids Res. 1989; 17(6): 2362 2468132 10.1093/nar/17.6.2362 PMC317610

[23] Ramakers C, Ruijter JM, Deprez RH, Moorman AF. Assumption-free analysis of quantitative real-time polymerase chain reaction (PCR) data. Neuro Sci Lett. 2003; 339(1): 62–66. . 12618301 10.1016/s0304-3940(02)01423-4

[24] Rieu I, Powers SJ. Real-time quantitative RT-PCR: design, calculations and statistics. Plant Cell. 2009; 21(4): 1031–1033. 10.1105/tpc.109.066001 19395682 PMC2685626

[25] Chen Y, Xiao-Man W, Li Z, Yi H, Dun W, Yan-Hua Q et al Rubisco Activase Is Also a Multiple Responder to Abiotic Stresses in Rice. PLoSONE. 2015; 10(10): e0140934 10.1371/journal.pone.0140934 PMC4610672 26479064

[26] Kurek I, Thom KC, Sean BM, Alfredo M, Lu L, Michael WL, Z. et al Enhanced thermostability of Arabidopsis rubisco activase improves photosynthesis and growth rates under moderate heat stress. The Plant Cell. 2007; 19: 3230–3241. 10.1105/tpc.107.054171 17933901 PMC2174701

[27] Ristic Z, Momcilovic I, Bukovnik U, Prasad PV, Fu J, Deridder BP, et al Rubisco activase and wheat productivity under heat-stress conditions. J Exp Bot. 2009; 60(14): 4003–4014. 10.1093/jxb/erp241 19671572

[28] Martinez-Barajas E, Molina-Galan J, Sanchez de Jimenez ES. Regulation of Rubisco activity during grain-fill in maize: Possible role of Rubisco activase. J Agri Sci. 1997; 128(2): 155–161.

[29] Morales A, Ortega-Delgado ML, Molina-Galan J, de Jimenez ES. Importance of Rubisco activase in maize productivity based on mass selection procedures. J Exp Bot. 1999; 50(335): 823–829. 10.1093/jxb/50.335.823

[30] Edae EA, Patrick FB, Harish M, Scott DH, Marc M, Marta SL et al Association mapping and nucleotide sequence variation in five drought tolerance candidate genes in spring wheat. The Plant Genome. 2013; 6(2). 10.3835/plantgenome2013.04.0010

[31] Whitney SM, Houtz RL, Alonso H. Advancing our understanding and capacity to engineer nature’s CO2-sequestering enzyme, Rubisco. Plant Physiol. 2011a; 155(1): 27–35. 10.1104/pp.110.164814 20974895 PMC3075749

[32] Carmo-Silva E, Salvucci ME. The regulatory properties of Rubisco activase differ among species and affect photosynthetic induction during light transition. Plant Physiol. 2013; 161: 1645–1655. 10.1104/pp.112.213348 23417088 PMC3613445

[33] Zhang N, Kallis RP, Ewy RG, Portis AR Jr. Light modulation of Rubisco in Arabidopsis requires a capacity for redox regulation of the larger Rubisco activase isoform. Proceedings of the National Academy of Sciences of the United States of America. 2002; 99: 3330–3334. 11854454 10.1073/pnas.042529999 PMC122518

[34] Portis AR Jr, Li C, Wang D, Salvucci ME. Regulation of Rubisco activase and its interaction with Rubisco. J Exp Bot. 2008; 59(7):1597–1604. 10.1093/jxb/erm240 18048372

[35] Kruger EL, Volin JC. Reexamining the empirical relation between plant growth and leaf photosynthesis. Functional Plant Biology. 2006; 33(5): 421–429. 10.1071/FP05310. 32689249

[36] Kumar A, Li C, Portis AR Jr. Arabidopsis thaliana expressing a thermostable chimeric Rubisco activase exhibits enhanced growth and higher rates of photosynthesis at moderately high temperatures. Photosynth Res. 2009; 100(3): 143–153. 10.1007/s11120-009-9438-y 19507049

[37] Yin Z, Meng F, Song H, Wang X, Xu X, Yu D. Expression quantitative trait loci analysis of two genes encoding Rubisco activase in soybean. Plant Physiol. 2010b; 152(3): 1625–1637. 10.1104/pp.109.148312 20032079 PMC2832260

