# Supplementary material for: The Expression of TaRca2-α Gene Associated with Net Photosynthesis Rate, Biomass and Grain Yield in Bread Wheat (Triticum aestivum L.) under Field Conditions
Source: PLoS One. 2016 Aug 22;11(8):e0161308. doi: 10.1371/journal.pone.0161308 (PMC4993480; doi:10.1371/journal.pone.0161308)
Supplement: S1 Fig — (DOCX) [file pone.0161308.s001.docx]

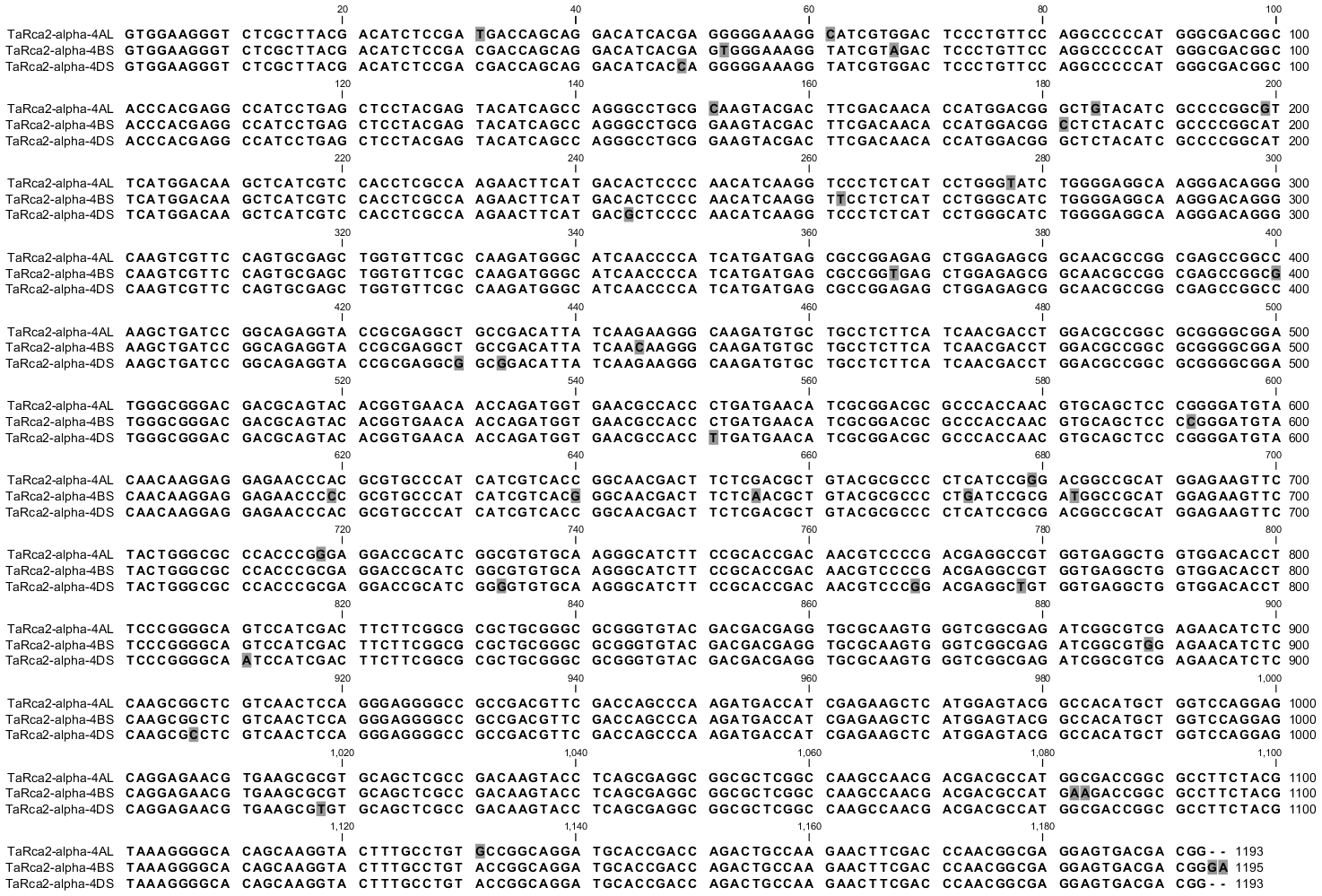


**Figure S1. CDS of *TaRca2-α-4AL*, *TaRca2-α-4BS and TaRca2-α-4DS*.** Shaded areas show nucleotides’

differences among the three homoeologous copies.
