# Supplementary material for: The Expression of TaRca2-α Gene Associated with Net Photosynthesis Rate, Biomass and Grain Yield in Bread Wheat (Triticum aestivum L.) under Field Conditions
Source: PLoS One. 2016 Aug 22;11(8):e0161308. doi: 10.1371/journal.pone.0161308 (PMC4993480; doi:10.1371/journal.pone.0161308)
Supplement: S2 Fig — (DOCX) [file pone.0161308.s002.docx]

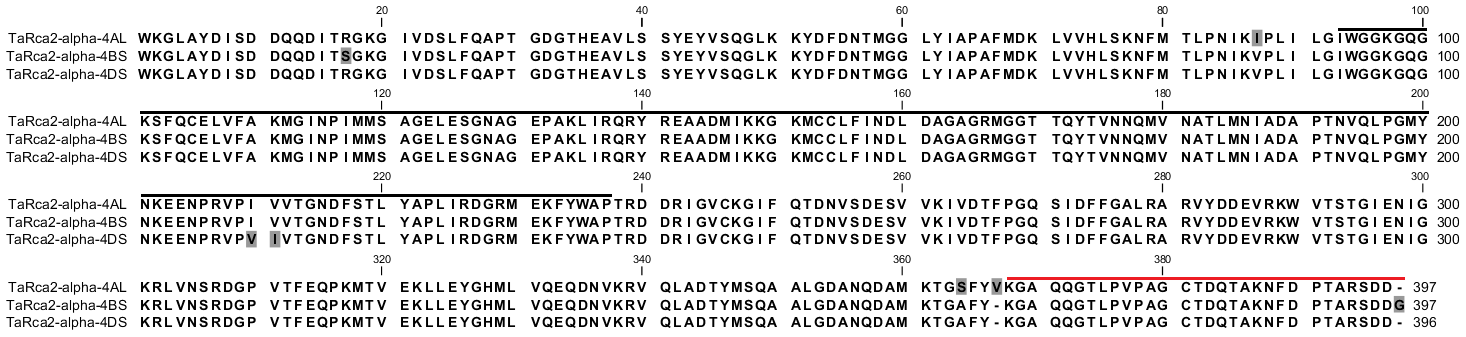


**Figure S2. Deduced amino acid sequences of *TaRca2-α-4AL*, *TaRca2-α-4BS* and *TaRca2-α-4DS*.** Black and red lines above the sequence show AAA and C-extension domains, respectively
