# Supplementary material for: The Expression of TaRca2-α Gene Associated with Net Photosynthesis Rate, Biomass and Grain Yield in Bread Wheat (Triticum aestivum L.) under Field Conditions
Source: PLoS One. 2016 Aug 22;11(8):e0161308. doi: 10.1371/journal.pone.0161308 (PMC4993480; doi:10.1371/journal.pone.0161308)
Supplement: S3 Fig — (DOCX) [file pone.0161308.s003.docx]

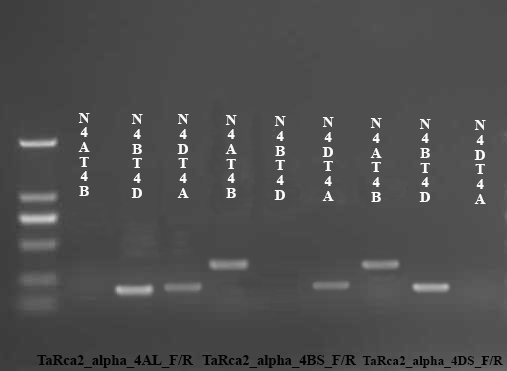


Amplicons produced by genome-specific primers (*TaRca2-α-4AL*, *TaRca2-α-4BS* and *TaRca2-α-4DS*) in nulli-tetrasomic (NT) lines of Chinese Spring (N4AT4B, N4BT4D and N4AT4A)
