# Supplementary material for: The Expression of TaRca2-α Gene Associated with Net Photosynthesis Rate, Biomass and Grain Yield in Bread Wheat (Triticum aestivum L.) under Field Conditions
Source: PLoS One. 2016 Aug 22;11(8):e0161308. doi: 10.1371/journal.pone.0161308 (PMC4993480; doi:10.1371/journal.pone.0161308)
Supplement: S1 Table — (DOCX) [file pone.0161308.s004.docx]

**Table S1 Correlation coefficients between the relative expression of the three copies of *TaRca2-α* at**

**heading (Z55), anthesis (Z67) and grain-filling (Z73) stages.**

|  | *TaRca2-α-4AL* (Z55) | *TaRca2-α-4BS* (Z55) | *TaRca2-α-4DS* (Z55) | *TaRca2-α-4AL* (Z67) | *TaRca2-α-4BS* (Z67) | *TaRca2-α-4DS* (Z67) | *TaRca2-α-4AL* (Z73) | *TaRca2-α-4BS* (Z73) |
| --- | --- | --- | --- | --- | --- | --- | --- | --- |
| *TaRca2-α-4BS* (Z55) | 0.851^**^ |  |  |  |  |  |  |  |
| *TaRca2-α-4DS* (Z55) | 0.854^**^ | 0.937^**^ |  |  |  |  |  |  |
| *TaRca2-α-4AL* (Z67) | 0.695^**^ | 0.696^**^ | 0.695^**^ |  |  |  |  |  |
| *TaRca2-α-4BS* (Z67) | 0.741^**^ | 0.793^**^ | 0.762^**^ | 0.696^**^ |  |  |  |  |
| *TaRca2-α-4DS* (Z67) | 0.757^**^ | 0.762^**^ | 0.741^**^ | 0.778^**^ | 0.900^**^ |  |  |  |
| *TaRca2-α-4AL* (Z73) | 0.724^**^ | 0.710^**^ | 0.707^**^ | 0.697^**^ | 0.709^**^ | 0.723^**^ |  |  |
| *TaRca2-α-4BS* (Z73) | 0.734^**^ | 0.708^**^ | 0.736^**^ | 0.685^**^ | 0.747^**^ | 0.758^**^ | 0.916^**^ |  |
| *TaRca2-α-4DS* (Z73) | 0.710^**^ | 0.716^**^ | 0.719^**^ | 0.705^**^ | 0.760^**^ | 0.755^**^ | 0.928^**^ | 0.954^**^ |

^**^ indicates correlation significant at the 0.01 level
